# Simulating cryo electron tomograms of crowded cell cytoplasm for assessment of automated particle picking

**DOI:** 10.1186/s12859-016-1283-3

**Published:** 2016-10-05

**Authors:** Long Pei, Min Xu, Zachary Frazier, Frank Alber

**Affiliations:** Molecular and Computational Biology, Department of Biological Sciences, University of Southern California, 1050 Childs Way, Los Angeles, CA 90089 USA

**Keywords:** Cryo-electron tomography, Particle picking, Macromolecular crowding, Visual proteomics

## Abstract

**Background:**

Cryo-electron tomography is an important tool to study structures of macromolecular complexes in close to native states. A whole cell cryo electron tomogram contains structural information of all its macromolecular complexes. However, extracting this information remains challenging, and relies on sophisticated image processing, in particular for template-free particle extraction, classification and averaging. To develop these methods it is crucial to realistically simulate tomograms of crowded cellular environments, which can then serve as ground truth models for assessing and optimizing methods for detection of complexes in cell tomograms.

**Results:**

We present a framework to generate crowded mixtures of macromolecular complexes for realistically simulating cryo electron tomograms including noise and image distortions due to the missing-wedge effects. Simulated tomograms are then used for assessing the template-free Difference-of-Gaussian (DoG) particle-picking method to detect complexes of different shapes and sizes under various crowding and noise levels. We identified DoG parameter settings that maximize precision and recall for detecting particles over a wide range of sizes and shapes. We observed that medium sized DoG scaling factors showed the overall best performance. To further improve performance, we propose a combination strategy for integrating results from multiple parameter settings. With increasing macromolecular crowding levels, the precision of particle picking remained relatively high, while the recall was dramatically reduced, which limits the detection of sufficient copy numbers of complexes in a crowded environment. Over a wide range of increasing noise levels, the DoG particle picking performance remained stable, but dramatically reduced beyond a specific noise threshold.

**Conclusions:**

Automatic and reference-free particle picking is an important first step in a visual proteomics analysis of cell tomograms. However, cell cytoplasm is highly crowded, which makes particle detection challenging. It is therefore important to test particle-picking methods in a realistic crowded setting. Here, we present a framework for simulating tomograms of cellular environments at high crowding levels and assess the DoG particle picking method. We determined optimal parameter settings to maximize the performance of the DoG particle-picking method.

**Electronic supplementary material:**

The online version of this article (doi:10.1186/s12859-016-1283-3) contains supplementary material, which is available to authorized users.

## Background

Cryo-electron tomography (Cryo-ET) has emerged as an effective tool for in-situ structural biology because it enables the imaging of macromolecular complexes in their native cellular environments at close to living conditions and at nanometer scale resolution [1–7]. In principle Cryo-ET can be used for studying the structure, abundance and spatial distribution of large macromolecular complexes in various cellular environments [8]. However, the simultaneous identification of all detectable macromolecular complexes in whole cell cryo-electron tomograms (i.e., visual proteomics) remains a considerable challenge. A visual proteomics approach would include the extraction of all potential complexes into individual subtomograms (i.e., particle picking) combined with large-scale reference-free subtomogram classification and subsequent averaging of subtomograms in the same class to generate density maps at increased resolution and signal to noise level [9–13]. However, extracting structural information from cell tomograms is very challenging due to several limitations, including the relatively low signal-to-noise ratio and distortions as a result of missing data (i.e., the missing wedge effect), which leads to a relatively low and anisotropic imaging resolution [5, 14–16]. Moreover, the crowded environment in cells makes the accurate identification and localization of macromolecular complexes an even more challenging task [2, 8, 9].

The first step in the analysis of macromolecular complexes in whole cell tomograms is an efficient and reliable automatic method for reference-free particle picking, namely the detection and extraction of subtomograms that likely contain individual macromolecular complexes. To perform realistic assessment and parameter optimization for particle picking in whole cell tomograms, one needs to first realistically simulate cryo-electron tomograms of crowded mixtures of macromolecular complexes. Although simulated subtomograms of isolated complexes have been used to validate template matching and sub-tomogram classification and averaging [8, 12, 16], simulated tomograms of crowded mixtures of macromolecular complexes have not been used to assess reference-free particle picking methods. Here we describe a systematic framework for simulating cryo-electron tomograms of crowded macromolecular mixtures, similar to those found in cell cytoplasm. Simulated tomograms were generated at various crowding and signal-to-noise (SNR) levels to perform an extensive assessment of the reference-free Difference-of-Gaussian (DoG) particle picking method [17]. To our knowledge no study exists to date that performed optimizations of parameter settings to maximize the accuracy for detecting likely locations of macromolecular complexes in crowded cellular tomograms. Our study specifically focused on the DoG performance for differently sized complexes of various shapes with respect to the cellular crowding and noise levels.

## Methods

This section is divided into two parts: In the first part, we describe the method for simulating tomograms of crowded cellular environments. In the second part, we describe how we assessed the DoG particle picking method on simulated tomograms at various crowding levels and signal to noise ranges.

### Simulating tomograms of cell-like environments

#### Generating cell-like environments

##### Selection of benchmark set

**Fig. 1:**
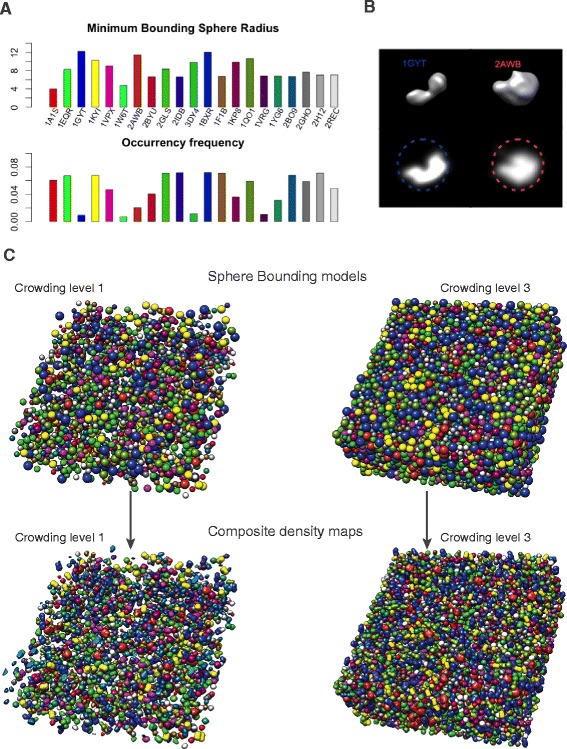
Framework for realistically simulating cryo-electron tomograms of crowded cellular environments. **a** The minimum bounding sphere radius (upper panel) and frequency (lower panel) for each of the 21 different types of macromolecular complexes in our benchmark set. Shown also are PDB ID of each complex (see Additional file 1: Table S1 for details) [18]. **b** Isocontour level representation (upper panels) and density plots (lower panels) of two complexes. The minimum bounding sphere of each complex enclosing each complex is also shown. **c** Crowded mixture of 2000 macromolecular complexes at 11 % (crowding level 1) and of 8000 macromolecular complexes at 44 % (crowding level 3) volume occupancy. Positions of spheres has been randomized and optimized to prevent sphere-sphere overlap. Each bounding sphere has been replaced by the corresponding complex’ randomly oriented density map. The composite density map serves as the input for simulating the cryo electron tomogram

To represent the crowded cellular environment we collected a total of 21 abundant macromolecular complexes of varying sizes and shapes from the Protein Data Bank (PDB) [18] (Methods, Fig. 1a). The electron optical density of a complex is proportional to its electrostatic potential, which is determined by its atomic structure [15, 19]. For each complex, density maps are generated at 4 nm resolution and with voxel size of 1 nm using the PDB2VOL program of the Situs2.0 package [20].

##### Generating crowded mixtures with random positions and orientations of all complexes

We then generated a composite density map of a crowded mixture of randomly placed and oriented complexes at high crowding levels, which mimicked the environment found in crowded cellular cytoplasm (Fig. 1). This density map then served as the input sample for simulating the cryo-electron tomography imaging process at different SNR levels.

To generate a crowded random mixture of complexes, we first represented each complex by its bounding sphere, which enclosed each complex. Then each complex was given a random copy number to define the composition of complexes in the mixture. After randomly positioning the corresponding spheres in a volume we used molecular dynamics simulations and simulated annealing for packing the crowded sphere mixture in a volume while preventing sphere-sphere overlaps. Then density maps of complexes were positioned in the corresponding spheres at a random orientation. The resulting composite density map of the crowded mixture was then used as the input sample to simulate the tomographic imaging of micrographs at different tilt angles followed by the reconstruction of the 3D density map to generate realistically simulated cryo-electron tomograms. These simulated tomograms contained imaging distortions from noise, missing wedge effects and effects from the Contrast Transfer Function (CTF). The computational details for each step are described in following subsections.

#### Minimum spherical bounding

We defined a minimum bounding sphere as the sphere with the smallest radius that entirely encloses the density map of the macromolecular complex at a given contour level. The contour level is a threshold to define a volume region of the complex [21]. We defined the contour level threshold as a proportion of the maximum density value in a density map. By inspection of the initial density maps for the 21 complexes, we empirically set the contour level ratio as *L* = 0.2, which resulted in a contour volume that best matches the van der Waals volume of the complexes. We then defined a subset of voxels (*R*) with density values larger than the contour level defined as:

$$ R\left(\boldsymbol{x}\right)=\left\{\forall \boldsymbol{x}\ \in\ {\mathrm{\mathbb{N}}}^{\mathbf{3}}\Big|D\left(\boldsymbol{x}\right)\ \ge\ Lm\right\} $$

Where *D*(***x***) with ***x*** = (*x*_**1**_, *x*_**2**_, *x*_**3**_) ∈ ℕ^**3**^ is the density map, *m* = max(*D*(***x***)|***x*** ∈ ℕ^**3**^) is the maximum density value of *D*, *Lm* is the contour level. *L* is the contour level ratio (i.e., the fraction of the maximum density value that defines the contour level).

Next, we calculated the convex hull for points located at all voxel locations with *D*(***x***) > **0** in *R* using the QuickHull algorithm [22]. The voxels in the interior of the convex hull regions were then used to calculate the minimum bounding sphere of the complex. The Emo Welzl’s algorithm was adapted to calculate the minimum bounding sphere for the set of voxels defined by the convex hull of the complex [23]. The minimum bounding sphere was used to simulate crowded mixtures of complexes. A minimum spherical bounding model has several advantages in comparison to other geometric bounding models such as cubic or cylinder models [24, 25]. The spherical bounding model is defined by only two descriptive parameters, the center and radius of the sphere, which simplifies the scoring function in the subsequent molecular dynamic simulations to minimize sphere-sphere overlaps. Also, in the subsequent replacement step, complexes can be placed at any random orientation within the sphere volume.

#### Generating macromolecular complex mixtures

The total volume occupancy of cell cytoplasms varies in different cells, and ranges between 5 % and 40 % in mammalian and between 34 % and 44 % in bacterial cells [26–29]. We defined the crowding level *C* as the ratio of the total volume occupied by all instances of macromolecular complexes and the total 3D volume of the tomogram.

$$ C=\frac{{\displaystyle {\sum}_{k=1}^n}{V}_k{N}_k}{V_T} $$

$$ {N}^{tot}={\displaystyle {\sum}_{k=1}^n{N}_k} $$

Where N_*k*_ is the copy number of macromolecular complex of type *k*, *N* ^*tot*^ is the total copy number of all complexes; *n* = 21 is total number of different types of macromolecular complexes, *V*_*k*_ is the volume of the k-th macromolecular complex type, which is estimated from region *R* defined in section 2.1.2 and *V*_*T*_ is the total volume of the tomogram defined by the length of its three principal.

In our study, each type of macromolecular complex is randomly assigned a copy number N_k_, following a multinomial distribution with parameter *N* ^*tot*^ and *f* = (*f*_1_, … *f*_*n*_), where *f*_*i*_ is a randomly selected frequency. We chose a random set of copy numbers because structures of many complexes and also their copy numbers in cells are still not known. It is challenging to determine the exact protein compositions in cells, which can differ even for the same cells under different growth conditions. To assess particle picking we therefore decided to have an entirely random mixture with variable sizes and shapes and copy numbers. Each instance of a macromolecular complex was also assigned a random orientation. To generate cellular environments at a defined crowding level we randomly positioned the bounding spheres of all complexes into a rectangular box volume. We then used molecular dynamics simulations and simulated annealing to optimize the packing of the crowded sphere mixture and remove any sphere-sphere overlaps. In our simulations the scoring function *S* ^*tot*^ consisted of two terms: First, a box volume restraint *S*_*i*_^*V*^, which enforced each sphere to lie within the volume of the simulation box, and second an excluded volume restraint *S*_*ij*_^*EX*^, which prevented any overlap between spheres.

$$ {S}^{tot}={\displaystyle \sum_{\mathrm{i}}^{{\mathrm{N}}^{tot}}}{S}_i^V+{\displaystyle \sum_{\mathrm{i}}^{{\mathrm{N}}^{tot}-1}}{\displaystyle \sum_{\mathrm{i}<j}}{S}_{ij}^{EX} $$

with

$$ {S}_i^V=\left\{\begin{array}{c}\hfill \frac{1}{2}{k}_d\mathrm{d}{\left(\mathrm{i}\right)}^2,\ \mathrm{if}\kern0.5em \mathrm{sphere}\ \mathrm{is}\ \mathrm{outside}\ \mathrm{the}\ \mathrm{container}\hfill \\ {}\hfill\ 0,\ \mathrm{if}\ \mathrm{sphere}\ \mathrm{is}\ \mathrm{inside}\ \mathrm{the}\ \mathrm{container}\kern0.5em \hfill \end{array}\right. $$

$$ {S}_{ij}^{EX}=\left\{\begin{array}{c}\hfill \frac{1}{2}{k}_p{\left[\mathrm{d}\left(\mathrm{i},\mathrm{j}\right)-\left({\mathrm{r}}_{\mathrm{i}}+{\mathrm{r}}_{\mathrm{j}}\right)\right]}^2,\ \mathrm{if}\ \mathrm{d}\left(\mathrm{i},\mathrm{j}\right)<\left({\mathrm{r}}_{\mathrm{i}}+{\mathrm{r}}_{\mathrm{j}}\right)\hfill \\ {}\hfill 0,\ \mathrm{if}\ \mathrm{d}\left(\mathrm{i},\mathrm{j}\right)>{\mathrm{r}}_{\mathrm{i}}+{\mathrm{r}}_{\mathrm{j}}\ \hfill \end{array}\right. $$

where *N* ^*tot*^ is the total number of spheres; *k*_*d*_ is the spring constant and *d*(*i*) is the smallest distance between the center of sphere i and the container border; *d*(*i*, *j*) is the distance between the centers of i-th and j-th spheres, *r*_*i*_, *r*_*j*_ are radius of the spheres. We used the IMP software package [30] to implement the scoring function and optimized the scoring function to a score of ~0. The initial velocities of all spheres were assigned based on a Maxwell-Boltzmann distribution at a given temperature. After starting from relatively high temperatures, an annealing process gradually reduced the temperature to relax the model.

$$ T(t)={T}_0-ct $$

Where *T*(*t*) indicates the system temperature at iteration step (time) t and *T*_0_ = 3000 is the initial temperature, *c* is a constant for gradually reducing the system temperature. We set *c* = 100. Finally a conjugate gradient optimization reduced the score to ~0. After generating crowded mixtures of spheres, we placed the randomly oriented density map of each complex into their corresponding bounding sphere. This procedure produced a composite density map of a crowded mixture of complexes. We generated several different density maps at various crowding levels (see below).

#### Generating simulated cryo-electron tomograms

For a reliable particle-picking assessment, cryo-electron tomograms must be generated by simulating the actual tomographic image reconstruction process, which allows for the inclusion of noise, tomographic distortions due to missing wedge effects, and electron optical factors such as Contrast Transfer Function (CTF) and Modulation Transfer Function (MTF) [8]. CTF and MTF describe distortions from interactions between electrons and the specimen and the distortions due to the image detector [8, 13, 31, 32]. The so-called missing wedge effect leads to image distortions due the limited the tilt angle range. A typical tilt angle range is ±60 or ±70 degrees, with step increments of 1 or 2 degrees [5, 33]. We follow a previously applied protocol and simulated 2D projection electron micrographs of our crowded macromolecular sample using a tilt angle range from -60 to 60 degrees with step increments of 2 degrees, which is a typical procedure for experimental tomograms [8, 13, 11]. For the simulated tomogram, we set typical acquisition parameters used in actual experimental measurements of whole cell tomograms: voxel size = 1 nm, the spherical aberration= 2 × 10^− 3^ m, the defocus value= − 4 × 10^− 6^ m, the voltage = 300 kV, the MTF corresponded to a realistic electron detector [34, 35], defined as sinc(πω/2) where ω is the fraction of the Nyquist frequency. Finally 3D tomograms were reconstructed via a back projection algorithm [11, 31] from 2D micrographs at various tilt angles.

Signal to noise ratio (SNR) is an important factor to control the level of distortions of a simulated tomogram [5]. The SNR was defined as the quotient of the variance of signal and the variance of noise [12].

$$ SNR=\frac{\sigma_{signal}^2}{\sigma_{noise}^2} $$

In the process of generating simulated tomograms, noise was added at two stages: one fraction was added to the signal before convolution with CTF and another fraction added after it was convoluted with CTF [12]. We simulated cryo electron tomograms at various SNR levels (i.e. SNR = [50,20,10,1]).

### Assessment of DoG particle picking

Our simulated tomograms of crowded mixtures of macromolecular complexes served as the ground truth for the assessment of the template-free Difference-of-Gaussian (DoG) particle picking method.

#### Background: Difference of Gaussian (DoG) filtering

A number of particle-picking methods have been proposed for cryo-electron microscopy images and adapted to cryo electron tomography [2, 8, 14, 17, 32, 36, 37]. Reference-based methods use information from a template in the search process to detect potential particle positions in the tomogram. Potential particle positions are detected as peaks in a cross-correlation function between the target tomogram and a template [2, 14, 32]. However, when the structure of a complex is unknown, reference-based methods cannot be applied. Unbiased visual proteomics approaches must rely on reference-free particle picking methods that are also applicable in the crowded environment of whole cell tomograms.

The reference-free DoG particle picking method is based on the Difference of Gaussian (DoG) image transform. A DoG map is created via subtraction of two versions of Gaussian filtered images and peaks detected in the DoG map are potential particles [17]. Previous studies tested the reliability of the DoG method for 2D cryo-EM images [17, 37]. However, no study exists that assessed the performance of the DoG method and performed parameter optimizations for reference-free particle picking in highly crowded tomograms of whole cells.

The Gaussian blurred map was obtained through a convolution of the Gaussian function *G*(*σ*) with the original map I and defined as:

$$ {I}_G\left(\sigma \right)=I\ast G\left(\sigma \right) $$

$$ G\left(\sigma \right) = \frac{1}{\sigma \sqrt{2\pi }}\ {e}^{-\frac{r^2}{2{\sigma}^2}} $$

Where *σ* is referred to as the scaling factor of the Gaussian function and r is the position vector in the image. A DoG map was built from subtracting two versions of the same map blurred through two Gaussian kernels with different scaling factors *σ*. The DoG map, for two different values of *σ*, was then defined as:

$$ {I}_{DoG}\left({\sigma}_1,{\sigma}_2\right)=I\ast G\left({\sigma}_1\right)-I\ast G\left({\sigma}_2\right) $$

In our study, we followed the DoG Picker design and defined the ratios between the two scaling factors as the k-factor.

$$ {\sigma}_2=k{\sigma}_1 $$

We set *k* = 1.1, which had been shown to be a reasonable value for applications in single particle cryo electron tomography [17]. We refer to *σ*_1_ as the DoG scaling factor and refer to it as *σ* from here on. The DoG scaling factor σ influences the performance of picking complexes of different sizes and the particle picking performance for different complexes will be evaluated for different scaling factors [17].

In our study, we first assessed the DoG particle picking performance with respect to different scaling factors, to identify an optimal setting. Then using the optimal scaling factor, we assessed the effects of noise and macromolecular crowding for the performance of the particle picking method.

#### Selection of local density peaks

To detect particle locations in a tomogram, we identified local density peaks in the DoG filtered tomograms (referred to as the set *P)* [38]. However, not all local density maxima correspond to complexes. Local density maxima can also result from noise. These maxima typically have lower density values than those of real complexes. We therefore used a lower density threshold *T* to define the set of local density maxima that likely correspond to particles *P*_*t*_. The density threshold *T* and the set *P*_*t*_ are defined as:

$$ T=m+t\cdot \frac{M-m}{K} $$

$$ {P}_t=\left\{v\ \in\ P\Big|D(v)\ \ge\ T\right\} $$

Where *M* is the maximum density value of all local maxima in *P*, *m* is the smallest density value for all local maxima, *K* = 20 is the number of bins, *t* = 0, 1, 2, …, *K* is the threshold level, *P*_*t*_ is the set of local density peaks at threshold level t, which had density values larger than the threshold *T*. In this paper we assessed the particle picking performance with respect to the threshold level *t* and determined the optimal value of *t* that maximizes the detection of complexes in the crowded environment.

#### Evaluating the particle picking performance

##### Assessment of true positives

To evaluate the particle picking performance, we need to determine correctly and falsely detected particles. We assume two conditions to define a true positive particle detection: First, the detected density peak should be close to the center of the true particle, i.e. the peak should be within a threshold radius from the true particle center. Second, we only count a true positive if only one local maximum is detected within the bounding sphere of the true particle. Multiple maxima within the bounding sphere would be counted as a false particle detection. Every local density peak can be assigned to at most one nearest particle.

To determine if a local density maximum is a true positive detection, we first defined the relative shift ratio *S* as the quotient of the distance between a detected local density peak to the center of its nearest particle and the radius of the minimum bounding sphere of the corresponding complex.

$$ S=\frac{\left|{x}_p-{C}_g\right|}{R_g} $$

Where *x*_*p*_ is the location of a local density peak, *C*_*g*_ and *R*_*g*_ are the center and radius of the minimum bounding sphere of its nearest complex. We set *S* ≤ 0.5 as a threshold to select local density peaks that are relatively near to the center of the ground truth complex. We can then determine how many particles are reliably detected with the DoG particle picking method.

##### Statistical Analysis of particle picking performance

Precision and recall is widely used as an assessment of information retrieval and is used to evaluate particle picking performances in cryo electron microscopy [8, 37]. The precision is defined as the fraction of the correctly detected versus all the detected peaks whereas the recall is defined as the fraction of the correctly detected peaks to the total number of particles in the ground truth dataset:

$$ precision = \frac{\#TP}{\#TP+\#FP} $$

$$ recall = \frac{\#TP}{\#TP+\#FN} $$

With #TP as the number of true positives, #FP is the number of false positives, and #FN is the number of false negatives in the particle detection.

In addition to precision and recall, we also use the F-score to evaluate the overall particle picking performance [37]. The F-score is defined as the harmonic mean of precision and recall.

$$ F- score = \frac{2\cdot precision\cdot recall}{precision+ recall} $$

By calculating the harmonic mean of precision and recall, we can compare the particle picking performance for different parameter settings and determine the optimal setting for a given tomogram.

## Results and discussion

In the following section, we first describe the set of simulated tomograms at various crowding and signal to noise levels. We then analyze the performance of the DoG particle picking method. Our goal is to assess the particle picking performance under varying parameter settings to determine the optimal conditions for particle picking in crowded environments. Then we evaluate the effects of noise addition and increasing cellular crowding levels on the performance of DoG based particle picking.

### Tomogram simulation

**Fig. 2:**
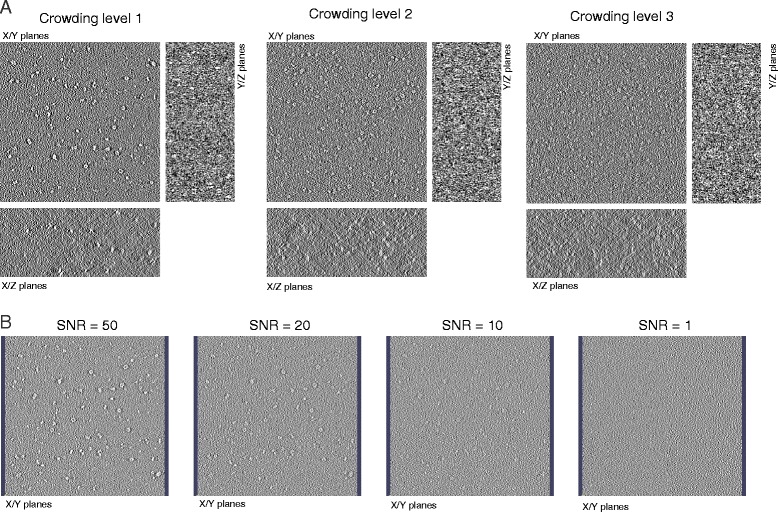
Simulated cryo-electron tomograms of crowded cellular environments at varying crowding levels and SNR levels. **a** XY, XZ, and YZ planes for simulated cryo electron tomograms of macromolecular complex mixtures at three different crowding levels containing 2000, 5000 and 8000 particles, respectively. Tomograms are simulated with a noise level of SNR = 50. **b** A single XY plane is shown for simulated tomograms with different SNR levels. Tomograms are shown for crowding level 1 containing a total of 2000 complexes

We selected 21 representative macromolecular complexes to generate a diverse mixture of complexes of variable sizes (Fig. 1, Additional file 1: Table S1). The particle sizes ranged from 79.2 to 245.2 Å in diameter. To simulate three different crowding levels in a cell-like environment, we generated mixtures of these complexes with randomly chosen copy numbers. Note, that in each of the three mixtures, a given type of complex has the same relative copy number frequency (i.e., the ratio of a complex’ copy number to the total copy number of all complexes). Macromolecular complex mixtures are generated containing 2000, 5000 or 8000 complexes in a 3D volume of 500 x 500 x 200 nm side length, which lead to cellular environments with crowding levels at 11 %, 26 % and 44 % volume occupancy, respectively (Figs. 1c and 2). These levels are comparable to crowding levels in bacterial and mammalian cells. At higher crowding levels, the macromolecular complex mixtures naturally occupy a higher fraction of the 3D volume and the average distance between adjacent macromolecular complexes is smaller (Fig. 2). This crowding effect is expected to have substantial influence on the DoG particle picking performance.

To study the influence of signal-to-noise (SNR) levels on the DoG particle picking performances, we choose different SNR levels ranging from SNR = 50, 20, 10 and 1. At lower SNR levels more noise is added to the tomogram (Fig. 2).

### DoG Particle picking assessment

#### Optimal scaling factor for DoG particle picking

Because the true locations and identities of all particles are known, the simulated tomograms serve as the ground truth to test the DoG particle picking and identify the parameter settings for optimal performance. Specifically, we tested settings for two parameters, the DoG scaling factor σ and the peak density threshold level *t* (Methods). Based on the sizes of typical macromolecular complexes (in our study the radius of macromolecular complexes ranges between 3-13 nm), we set *σ* to be the following set of values [3, 5, 7, 9, 11, 13] in nm units. The density threshold *t* ranged between 0 and the maximal value *K* = 20 and determined the minimum density value at which a local maximum is considered as a predicted particle location. Local maxima with voxels density values larger than the cutoff *t* were considered as predicted particle positions.

**Fig. 3:**
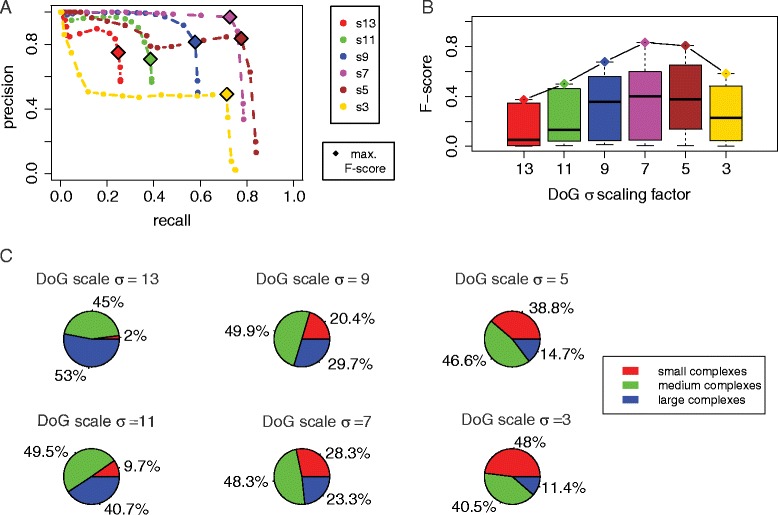
Assessment of DoG particle picking with different scaling factors. **a** Precision and recall curves for DoG particle picking for different scaling factors (colored curves), applied on tomograms at crowding level 1 (2000 particles) with SNR = 50. Each point defines the recall and precision using a different density threshold *t*. Optimal F-score performance is shown by a diamond. **b** Boxplot of F-score distributions for particle picking at different threshold levels *t* for each DoG scaling factor. The diamond shaped points show the best F-score for each scaling factor. **c** The impact of particle size on the DoG performance with different scaling factors. The pie charts indicate the proportions of correctly predicted particle locations for complexes of different sizes (small, medium, and large complexes) for particle picking with different DoG scaling factors

We first performed the analysis on tomograms with a crowding level of 11 % (2000 particles) and SNR = 50 (Fig. 2). To illustrate the performance of particle picking, we calculated a precision-recall (PR) curve, by determining for each *t* threshold value the corresponding precision and recall (Fig. 3). A PR curve was calculated for each of the scaling factors σ. With increasing threshold cutoff *t*, detected peak positions must have larger density values to be considered as particles. As expected, the precision increased with increasing *t* values for all σ values, however, the recall dropped considerably with increasing *t* values and a smaller amount of particles were successfully detected (Fig. 3). With a threshold cutoff *t* = 0, the maximum recall for each scaling factor was reached (Fig. 3).

Large differences were observed when comparing the PR curves for different σ values (Fig. 3). The poorest performance was observed for the smallest and largest σ values (*σ* = 3 and *σ* = 13), whereas the best performance was observed for *σ* = 5 and *σ* = 7. At large scaling factors, the recall was especially poor. For example at *σ* = 13 the maximum recall reached only 25.6 % due to the relatively small number of detected local maxima. Only a total of 900 local maxima were detected in the DoG map, even if all peaks were considered (at *t* = 0). This observation indicates that for *σ* = 13 the locations of many complexes, especially those of smaller sizes, do not coincide with detectable local maxima in the DoG map. For *σ* = 11 the recall increased to 38.6 %. For comparing the overall performance, we determined for each parameter setting the maximal F-score, which is the harmonic mean of the precision and recall (Methods) (Table 1). The best performance overall was observed for *σ* = 7 and t = 3 with a maximal F-score of 0.831, and a precision of 96.9 % and a recall of 72.7 %, which indicated that DoG particle picking performed well in terms of both accuracy and completeness.

**Table 1:** Particle picking performance and parameter settings for optimal particle picking performance (maximal F-score)

Sigma	Optimal *t*	Precision	Recall	Best F-score	# Local maxima (at *t* = 0)	# Local maxima (optimal *t*)
13	3	0.659	0.256	0.373	900	775
11	2	0.709	0.386	0.499	1378	1088
9	2	0.813	0.578	0.676	2341	1421
7	3	0.969	0.727	0.831	4661	1500
5	3	0.837	0.777	0.806	12741	1856
3	5	0.492	0.714	0.583	66180	2903

The selected scaling factor had large impact on the performance, and also showed that a smaller scaling factor not always performed better. Very large and small scaling factors decreased the performance. The most dramatic loss of precision was observed for very small sigma values (*σ* = 3). With *σ* = 3, a very large number of false positive local maxima was detected. In summary, we conclude that the optimal DoG scaling factor is *σ* = 7 for detecting macromolecular complexes in crowded cellular environments. The performance for a given *σ* value is expected to be affected by the particle sizes (Fig. 3). In the next section we analyze the impact of particle size on the performance.

#### Size specificity of DoG particle picking

To test the DoG performance for particles of different sizes, we categorized the complexes into 3 groups (small, medium and large complexes) and tested the DoG particle picking performance for different scaling factors separately for each group. Complexes with a bounding sphere radius smaller than 7 nm were defined as small complexes, the complexes with bounding sphere radius between 7 and 10 nm were defined as medium-sized complexes and the remaining complexes were defined as large complexes. For each scaling factor, we determined the fraction of correctly predicted complexes in each group of complexes (using the *t* values leading to the maximal F-score).

With large scaling factors *σ* = 13, 11, only a very low proportion of small complexes were among the detected true positives (Fig. 3). With smaller scaling factors *σ* = 9, 7, 5, 3, this proportion increased and gradually became a major component of all detected particles. This observation confirmes that a specific scaling factor targets a certain size of particles. The most balanced performance over all complex sizes is observed with the scaling factor of *σ* = 7. Interestingly, medium sized complexes were detected correctly at relatively high fractions across all the σ values, whereas smaller and larger complexes were only detected with small and large σ values, respectively. We confirm that there is an optimal scaling factor that performed well for a given complex size.

**Fig. 4:**
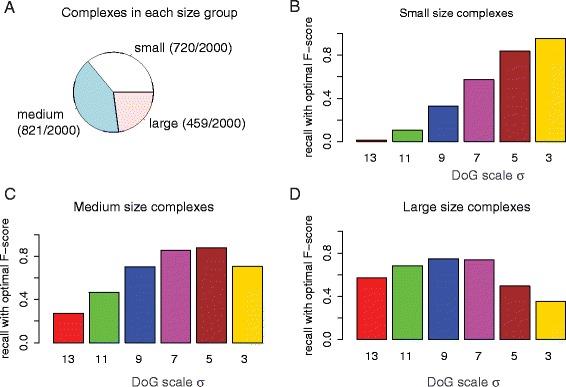
Size specificity of DoG particle picking. **a** Fraction of macromolecular complexes in each size group. **b**-**d** Recall for complex detection with varying scaling factors (using the threshold level to achieve best F-score) for the groups of small, medium-sized and large complexes, respectively

We then compared how strongly the recall was affected when the scaling factors were varied (Fig. 4). The most dramatic changes in recall upon variation of sigma values were observed for the group of small complexes. Whereas small sigma values produced excellent recall, extremely poor recall were observed when using larger *σ* values (Fig. 4b). In contrast, for the group of large complexes, the recall remained similar over a wider range of σ values, with the lowest recall observed for the smallest *σ* value (Fig. 4d and Table 2). Most efficient detections of macromolecular complexes tended to be achieved by applying a DoG scaling factor in accordance with the target complex size. Our observations indicate that a single *σ* value in DoG particle picking is not the best option for visual proteomics approaches when target complexes have largely varying sizes. In the next section we discuss the strategy for combining multiple *σ* values to enhance overall performance in particle picking.

**Table 2:** Group sized particles recall with optimal particle picking (maximal F-score)

Sigma	Small	Medium	Large
13	0.043	0.395	0.614
11	0.182	0.561	0.697
9	0.467	0.754	0.780
7	0.720	0.909	0.839
5	0.836	0.858	0.624
3	0.649	0.579	0.413

#### Multiple size particle picking

**Fig. 5:**
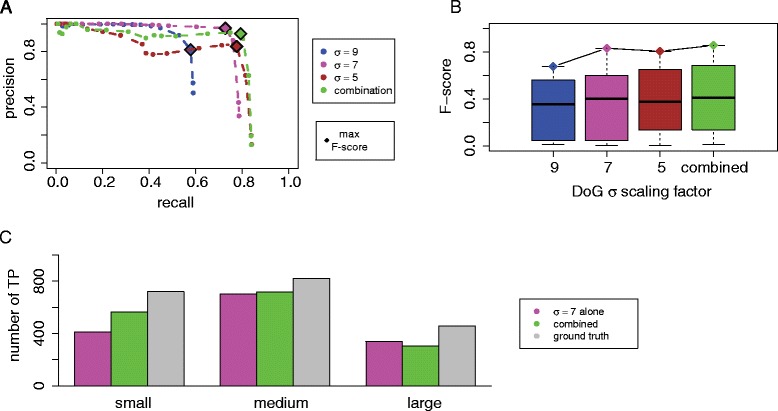
Evaluation of combination strategy for particle picking. **a** Comparison of precision and recall curves when using individual scaling factors or a combination of scaling factors in particle picking for tomograms (crowding level 1, SNR = 50). **b** Comparison of F-scores for particle picking with individual or a combination of scaling factors. **c** Comparison of the number of true positives detected in particle picking when using the single optimal scaling factor (*σ* = 7) and a combination of scaling factors (*σ* = 7, 5, 9) for complexes of different size. The barplot shows the number of true positives (reliably detected particles) and the ground truth copy numbers for differently sized complexes (small, medium, large)

Our observations confirm that the performance of DoG particle picking at a given sigma value is sensitive to the size of the target complex. Here we provide a strategy to optimize the detection of particles of variable sizes. Since a given scaling factor *σ* performs better for particles of a certain size, we searched for all local density peaks detected with different σ values (*σ* = 7, 5, 9) and filtered out peak overlaps. We applied the DoG peak detection in sequential order, using first σ values with the highest F-score before those with lower F-scores. We first used the scaling factor *σ* = 7, which showed optimal overall performance in our study, followed by peak detection with scaling factors 5 and 9. We defined an overlap between two peaks if the peak-peak distance is smaller than 7 nm. If two peaks are closer than this value then we select only one of the two peaks, namely the peak location determined by the scaling factor with the higher F-score (i.e., *σ* = 7) and removed the redundant peak. As shown in Fig. 5, using this combined approach we were able to enhance the recall for the groups of small and medium sized complexes. The precision was slightly reduced in comparison to the performance for scaling factor *σ* = 7. However, the F-score was improved for all particle sizes. We conclude that the combination strategy detects more particles of varying sizes with acceptable high precision.

#### Crowding and SNR level effects

**Fig. 6:**
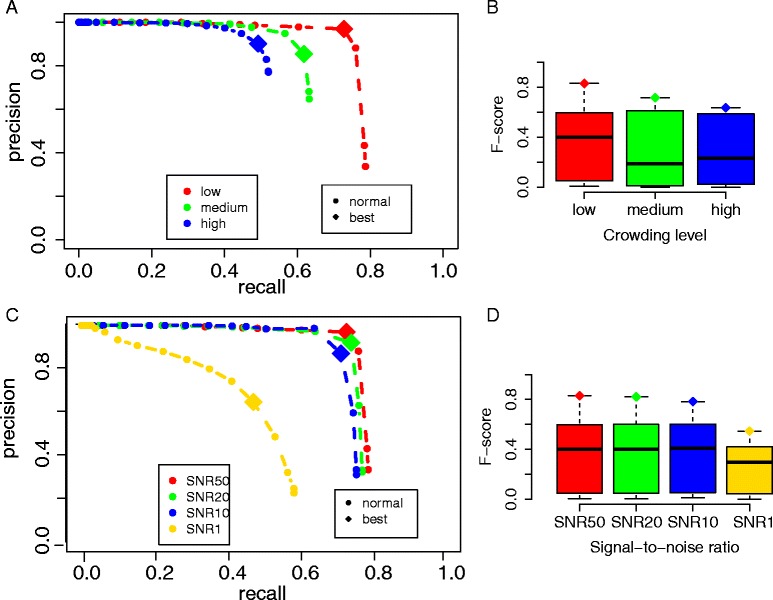
Evaluation of different crowding levels and SNR levels. **a** Precision and recall curves of particle picking at different crowding levels using a scaling factor of 7 (low = crowding level 1 with 2000 particles, medium = crowding level 2 with 5000 particles, high = crowding level 3 with 8000 particles), and tomograms with SNR = 50. **b** F-scores for particle picking across all threshold levels *t* and scaling factor 7, grouped by different crowding levels. **c** Precision and recall curves for particle picking at different SNR levels, using low crowding level 1 (with 2000 particles) and scaling factor 7. **d** F-scores for particle picking across all threshold levels *t* and scaling factor 7, grouped by different SNR levels

Naturally, detection of the positions of macromolecular complexes should be easier when particles are more sparsely distributed. Therefore crowding levels could affect the particle picking performances. In highly crowded cellular environments, macromolecular complexes can be so close to each other that it may become challenging to distinguish adjacent complexes. Figure 6 shows the performance with the optimal scaling factor *σ* = 7 under different crowding levels, ranging from volume occupancy of 11 %, 26 % to 44 %. As expected, the maximum recall of 78.7 % was observed at the lowest crowding level. The recall consistently decreased with increasing crowding levels, reaching 63.2 % for medium and only 52.0 % for high crowding levels (Fig. 6). The maximal F-score also decreased from 0.831 to 0.717 at medium crowding and to 0.637 at the highest crowding level (Tables 3 and 4). Finally, we also investigated the level of noise on the particle picking performance. We generated tomograms at four different noise levels, ranging from SNR = 50, 20, 10 and 1 (Fig. 6). As expected, the SNR level had large influence on the DoG particle picking performances. For tomograms at SNR = 50 and scaling factor σ = 7, the DoG particle picking achieved high precision and recall. Although the particle picking performance became generally less effective with decreasing SNR, the performance remained relatively stable over a wide range of SNR levels (SNR = 50,20,10) with the maximal recall ranging from 78.7 % (SNR = 50), 77.0 % (SNR = 20) and 75.5 % (SNR = 10) (Fig. 6). However, at SNR = 1 the maximal recall drops dramatically to <60 %. The maximal F-score remained at around 0.8 over a wide range of SNR (SNR = 50, 20, 10) and dropped to 0.546 at SNR = 1 (Tables 3 and 4). We conclude that despite the good performance of DoG over a wide range of SNR levels, the DoG performance can drop abruptly if SNR levels are below a certain boundary.

**Table 3:** Crowding levels and optimal particle picking (maximal F-score)

Crowding	Optimal t	Precision	Recall	Best F-score
11 %	3	0.969	0.727	0.831
26 %	2	0.855	0.618	0.717
44 %	3	0.902	0.492	0.637

**Table 4:** SNR levels and optimal particle picking (maximal F-score)

SNR	Optimal t	Precision	Recall	Best F-score
50	3	0.969	0.727	0.831
20	3	0.921	0.740	0.821
10	3	0.871	0.712	0.783
1	5	0.649	0.472	0.546

## Conclusions

In this study, we assessed DoG particle picking using realistically simulated tomograms of simulated crowded cell cytoplasm. Automated and reference-free particle picking is an important first step in a visual proteomics analysis of whole cell tomograms. It is therefore important to test the performance of the DoG method for particles of variable size, under different crowding and noise levels. To achieve this goal, we first proposed a framework for realistically simulating Cryo-ET tomograms of cellular environments at different crowding levels. Our approach used a minimum bounding sphere model and molecular dynamics to generate crowded mixtures of macromolecular complexes. The simulated tomograms served as a ground truth dataset for evaluating the reference-free DoG particle picking method. Taking both accuracy and completeness into consideration, we used precision and recall to statistically evaluate how well particles can be detected with different DoG scaling factors. Our benchmark included complexes of different sizes and shapes. For these complexes, DoG performs best with medium sigma values. For instance the scaling factor *σ* = 7 with a threshold value *t* = 3 lead to the best F-value among all tested scaling factors. With very large scaling factors (i.e. *σ* = 13), the recall was very poor and only a small number of particles could be detected. Similarly, very small scaling factors (i.e. *σ* = 3) underperformed and lead to the lowest observed precision among all scaling factors. However, as expected the scaling factor performance depended on the complex size. When complexes were small, smaller sigma values performed better. For instance σ = 3 lead to the best recall for small complexes, while *σ* = 3 lead to very poor performance for medium and large complexes. We then proposed an iterative strategy to combine different DoG settings to maximize the overall performance of the DoG particle picking for visual proteomics settings, where one expects to detect complexes of variable sizes. Finally, we concluded that both macromolecular crowding and SNR influences the DoG particle picking performances. Tomograms with highly crowded cellular environments and particularly very high noise levels (low SNR) can make it challenging to accurately detect macromolecular complexes.

## Additional file

Additional file 1: The benchmark set of macromolecular complexes. The PDB ID, PDB name, molecular weights, minimum bounding sphere radius, occurrence frequencies and copy numbers for each crowding level of the macromolecular complexes used in this study. (XLSX 10 kb)

## Abbreviations

Cryo-ET: Cryo-electron tomography
CTF: Contrast transfer function
DoG: Difference of Gaussian
MTF: Modulation transfer function
PDB: Protein data bank
SNR: Signal to noise ratio.
